# Adolescent Idiopathic Scoliosis: Advances in Diagnosis and Management

**DOI:** 10.1007/s12178-024-09939-2

**Published:** 2024-12-30

**Authors:** Alexander H. Jinnah, Kyle A. Lynch, Taylor R. Wood, Michael S. Hughes

**Affiliations:** Wake Forest School of Medicine, Department of Orthopaedic Surgery, 1 Medical Center Blvd, Winston Salem, NC 27103 USA

**Keywords:** Scoliosis, Management, Diagnosis

## Abstract

**Purpose of Review:**

Adolescent idiopathic scoliosis (AIS) is a disabling spinal pathology, with a significant morbidity if left untreated. This review investigates the recent advances in the diagnosis and management of AIS.

**Recent Findings:**

Low radiation techniques have become a paramount focus in the management of patient’s with AIS. The EOS system is growing in popularity due to lower radiation compared to conventional radiographs with the added advantage allowing 3D reconstruction. Bracing remains the standard treatment for curves amenable to this, however, due to the importance in compliance with brace wear there has been a recent push for more personalized brace construction using 3-dimensional printing. If curves are not amenable to bracing, then surgical intervention is generally recommended. Posterior spinal fusion (PSF) remains the gold standard; however, newer growth modulating techniques are in their infancy. Anterior vertebral body tethering (VBT) is a relatively novel method of treatment for AIS, that has seen promising early results. Due to its novelty and varying results VBT use remains limited and will need to be further investigated.

**Summary:**

AIS is a complex disease without a clear understanding of it’s etiology. If identified earlier, then non-operative treatment may lead to prevention of curve progression and the need for surgical intervention. Newer technologies, such as the EOS system, allow 3D reconstruction of curves which can assist with pre-operative planning. PSF remains the gold standard surgical intervention for AIS, however, new developments in alternative techniques could have a promising future, especially for skeletally immature patients.

## Introduction

### Embryology

Adolescent idiopathic scoliosis (AIS) is a disabling spinal pathology that manifests in children and adolescents and is characterized by an S-shaped spinal deformity, rib hump, and chest curvature. Despite AIS being one of the most common pathologies causing disability in children, its etiology remains unknown. Researchers have speculated that the origin of scoliosis may involve adipocytes, osteoblasts, myoblasts, and more recently visceral, white adipose tissue that lead to asynchronous neuro-osseus growth involving the anterior and posterior columns of the vertebrae and the spinal cord [1–3]. Given that these cell types involve different embryological origins, including mesenchymal stem cells, the somatic myotome, and lateral plate mesoderm, it is suggested that these alterations may originate from a process during fetal growth [1, 2]. More recently, Zaydman et al. [4] showed that altered neural crest cell migration involving Pax3 gene expression may be an embryologic etiology of AIS, therefore opening the door for further exploration of genetic or epigenetic causes of AIS.

### Development

Any discussion of AIS must take into account both the historical and current perspectives regarding the natural history of untreated AIS. For the surgeon managing AIS, the difficulty lies in being able to predict the future outcome of a curve both with and without intervention. This prediction relies primarily on a few long-term cohorts of patients with major curves that were left untreated. While limited by their retrospective nature and the inclusion of mixed etiology scoliosis, landmark papers by Nilsonne [5] and Nachemson [6] out of Sweden in 1968 began to show a pattern of early death, disability, and cardiopulmonary compromise in major curves that were left untreated [7]. Perhaps the most important insights into the natural history of AIS come from the long-term cohorts out of Iowa, which followed patients for an average of 40.5 years and showed that, in general, curves ≤ 30° at maturity tended not to progress, while curves between 50–75° tended to progress the most [8]. Among those with progression, the Iowa cohort also showed that pulmonary deficits were directly correlated with curve size, although the significant mortality and disability found in the Swedish cohort was not redemonstrated [9]. They additionally found that patients with untreated AIS experienced more chronic and acute episodes of back pain than their peers, however this did not limit their ability to work or perform activities of daily living [9]. Based on these cohorts and a multitude of other factors, international societies formulated treatment recommendations which can be used by the clinician to help guide treatment decisions [10].

### Pathogenesis

There are a multitude of theories regarding the pathogenic cause of AIS, therefore the etiology is now considered multifactorial. Since an important component of AIS is rotational deformity, it has been postulated that asymmetrical ossification of the neurocentral cartilage results in asymmetrical pedicle growth and therefore rotational abnormality [11]. The re-emergence of the emphasis on sagittal plane deformity, in addition to the coronal plane, in AIS has led to the assumption that generalized anterior column overgrowth in the spine is implicated as well [11]. Growth factors may also be involved given the increased growth rate near puberty, which may alter the ability to maintain posture and balance and lead to curve progression in AIS patients [11]. Decreased melatonin levels have also been associated with scoliosis in animal models, but after a lack of translation to humans, focus has turned toward increased calmodulin (melatonin antagonist and actin-myosin complex regulator in skeletal muscle) levels in those with AIS [4, 11]. The biomechanical and biochemical implications of lower bone mineral density, wedge-shaped vertebral bodies, and alteration of vertebral disc shape are other proposed mechanisms of curve progression as well [4, 11]. Finally, there is a genetic component to AIS given familial clustering shown in twin studies and AIS patient pedigree analysis, however decades of research have failed to isolate reproducible genetic alterations [4, 11]. More recent studies are now turning focus toward epigenetic causes and alterations in neural crest cell migration with promising hypotheses to explore [4, 12].

### Prevalence

The prevalence of AIS is thought to be between 0.47—5.2%, with a female to male range of 1.5:1—3:1 [13]. In curves > 40◦, the female to male ratio increases up to 7.2:1 [13]. Of note, most estimates of AIS prevalence are taken from school screening studies in countries around the world with differing methodologies, however results tend to be consistent across most estimates.

## Diagnosis

### Screening

Historically scoliosis screening has been performed in school age children [14]. However, in 2004 the Unites States Preventive Services Task Force (USPSTF) changed their recommendations from insufficient evidence to make a formal recommendation (“I”) to a recommendation against screening (“D”) [15]. This was due to lack of evidence demonstrating that screening led to an increase in diagnosis of AIS. This reverted back to an “I” recommendation in 2018 due to a position statement released by the Scoliosis Research Society (SRS), Pediatric Orthopaedic Society of North America (POSNA), American Academy of Orthopaedic Surgeons (AAOS), and the American Academy of Pediatrics [15, 16].

The most commonly performed screening method is the Adam’s Forward bend test (FBT), with or without scoliometer measurements [17, 18]. The Adam’s FBT is the simplest screening test and consists of the patient standing facing away from the examiner, the patient then bends at the waist until the spine is parallel to the floor. At this time the examiner will assess the trunk for asymmetry of the back, ribs, and shoulders. If any asymmetry is noted, this is considered a positive test [16]. This is often combined with the use of a scoliometer, which quantifies the degree of spinal rotation [15]. This instrument is placed on the patient’s back while they are performing the FBT at the point of maximal asymmetry. A reading greater than 5–7 degrees is usually used to identify patients that need further evaluation [15, 16]. Moiré Topography is another screening method that has been used [15]. This consists of using a lighted imaging device to project a series of lines onto the patient’s back. The lines are then distorted by the contours of the patient’s back, creating a three-dimensional map. This map is then photographed, and can be evaluated for asymmetric markings, which are termed “Moiré fringes”. If more than 2 Moire fringes are present, this leads to a referral to a specialist [19]. The disadvantages of the Moire topography include the additional cost of equipment and a high false positive rate [20–22]. In Hong Kong a two tiered voluntary system is used, in which first screening is done using the FBT with scoliometer, and if positive these patients then underwent the second tier using Moiré topography [15]. Two studies demonstrated this two tiered screening system yielded a 99.2% sensitivity, 93.8% specificity, 0.8% false positive rate, 6.2% false negative rate, and a positive predictive value of 81% [23, 24].

### Diagnosis

Once patients have undergone screening, radiographs are recommended for the definitive diagnosis of AIS [25]. Standard radiographs include upright standing posterior-anterior (PA) and lateral views of the entire spine [25, 26]. On the PA radiographs the degree of the largest curve is measured using the Cobb method [27]. The lateral views will be used to identify any sagittal abnormalities [25]. Bending films may be obtained to determine flexibility of the curve [25]. Additionally, magnetic resonance imaging (MRI) is obtained for patients that have non-classical presentations, such as left sided thoracic curve, neurologic deficiency, cavus feet, or unexplained headaches [25, 26].

Once diagnosed, these patients have repeated radiographs and thus radiation exposure throughout these children’s lives. For this reason, the importance of low radiation techniques has become a paramount focus [26, 28]. The EOS slot-scanning 2D/3D system has 50–80% lower radiation compared to conventional radiographs [28]. This technique consists of 2 orthogonal x-ray sources simultaneously allowing acquisition of 2 images over about 10–15 s. [29] This therefore provides the added advantage of biplanar imaging allowing 3D reconstruction of the deformity [28–30].

### Management

Treatment options for AIS once it is diagnosed include observation, exercises, bracing, and surgery [14, 31–36]. The treatment option chosen is reliant on the risk of spinal curve progression, which is increased with a higher Cobb angle and lower Risser grade [14]. Observation is generally accepted for patients with a Cobb angle less than 20⁰−25⁰, with repeat radiographs every 4–6 months [37–40]. If there is progression noted in the curve the patient should then be evaluated for further treatment, such as bracing or surgical intervention [37, 38, 40].

#### Bracing

Bracing has become a mainstay in the treatment of AIS with the publication of the important studies by Nachemson and Peterson [41], Weinstein [38], and Danielsson [42]. Nachemson and Peterson [41] demonstrated that the use of bracing had a success rate in preventing curve progression of at least 6⁰ in 74% of patients, compared to 34% with observation alone. Danielsson et al [42] found that at 16 year follow up none of the patients that were braced required surgery, whereas 10% of the patients in the observation group had. However, the work by Nachemson [41] and Danielsson [42] were both nonrandomized cohort studies, therefore Weinstein et al [38] underwent the Bracing in Adolescent Idiopathic Scoliosis Trial (BRAIST), a multicenter randomized controlled trial. The BRAIST study included patients 10–15 years old, risser stage 0–2, with curves measuring 20⁰−40⁰. The patients were randomized to either wear a rigid thoracolumbosacral orthosis (TLSO) for 18 h/day or observation alone. The study found that 72% of patients who wore the brace had curves < 50⁰ at time the study was completed or they reached skeletal maturity, versus 48% of the observation group. Additionally, if patients wore their brace for ≥ 12.9 h/day the success rate was 90–93%. In the BRAIST study a temperature-sensitive probe was used in order to monitor compliance and has been advocated for, due to the importance identified with this [37, 38].

It is important to note that there are several different brace designs available. The Boston brace system was first designed in 1972 by Marion Miller and John Hall at Boston Children’s Hospital [43]. The principles behind the Boston Brace are a standardized or custom made symmetric module, a brace blueprint, lumbar and pelvic fixation, active and passive curve correction, pad pressure at the apex and below the curve, and pressure relief opposite to every area of force [37]. The brace itself is made using computer aided design software in conjunction with the patient’s measurements. Following the fabrication of the brace, custom pads may be positioned at the apex and below the curve on the convex side. Additionally, contralateral relief windows are made in the brace structure. The Boston Brace is most commonly used in patients with curves with an apex between T8 and L2, and has seen correction and control of these curves in several studies [37, 41, 44].

The Providence bracing system is a newer brace that was developed in 1992 by Charles d’Amato and Barry McCoy in Providence, Rhone Island [45]. This brace was designed to be worn at night, with a focus on derotation and lateral vectors for curve correction [37]. This particular design has been used more often in mild curves (< 35⁰) [37]. The data regarding the effectiveness of the Providence brace in comparison to the Boston brace is varying, and thus both are considered to be effective treatments for AIS [46].

Another bracing system that is used is the Rigo-Cheneau brace, which is a modification of the original Cheneau front-opening brace [37]. The original brace provided three-dimensional correction of spinal curves using convex to concave pressure points. This design was then modified by Dr. Rigo to adjust the curve pattern to that of the patient. The adjusted design has four types of patterns, which include imbalanced thoracic, double curve, balanced thoracic, and false double, and single lumbar or thoracolumbar [47]. The Rigo-Chembeau brace has been successful in achieving curve control, especially in older patients with smaller curves [48].

Due to the importance outlined earlier in compliance with brace wear there has been a recent push for more innovative and personalized brace construction using 3-dimensional printing technology [49]. The thought behind using this technology is that a custom brace can be created with ideal biomechanical effectiveness and would be more comfortable, thus encouraging better compliance. It is important that randomized controlled trials be carried out to evaluate the effectiveness of these, and there are currently two such studies underway [49, 50].

#### Operative Treatment

If scoliotic curves progress despite conservative treatment or the curve exceeds 40–50◦ in an immature skeleton, this warrants surgical intervention [26, 51, 52]. The goals of surgical intervention are stopping curve progression, optimizing spinal balance, improving cosmesis, and minimizing pain and morbidity [51, 53]. Surgical options include spinal fusion and newer growth modulating techniques [51, 52].

Spinal fusion surgery has been used for over 100 years, with anterior spinal fusion (ASF), posterior spinal fusion (PSF), or a combined anterior and posterior approach [26, 51]. Studies have demonstrated that there is no difference in blood loss, length of hospital stay, and patient outcomes between anterior and posterior approaches [26]. However, some theorize that a posterior approach may save on negative effects that the anterior approach will have on pulmonary function [26]. Thus, PSF remains the gold standard. However, PSF does involve an extensive muscle dissection and require transfusion in 18–30% of patients [54–56]. Additionally, PSF limits further longitudinal spinal growth, and decreases spinal range of motion. Thus, there has been continued advancement of procedures to include fusionless growth modulating techniques [36, 51].

Anterior vertebral body tethering (VBT) is a relatively novel method of treatment for AIS, that has had some promising early results [57–59]. This technique takes advantage of the “Hueter-Volkmann Law”, which suggests that altering the mechanical environment of the vertebral physis will allow guidance of the vertebral growth [60, 61]. This technique consists of tethering the spine using screw instrumentation with a tether connecting the screws together via an anterior thorascopic approach [51, 62]. Due to its novelty and varying results the use of VBT is only allowed under the Humanitarian Device Exemption (HDE) from the United States Food and Drug Administration (FDA) [63]. The HDE states, “This device is indicated for skeletally immature patients that require surgical treatment to obtain and maintain correction of progressive idiopathic scoliosis, with a major cob angle of 30 to 65 degrees whose osseous structure is dimensionally adequate to accommodate screw fixation, as determined by radiographic imaging.” Some authors that have published they use, which remain very limited and include skeletally immature Lenke type 1 patient with Cobb angles roughly from 40⁰−65⁰ [51, 59]. There are many unknown factors associated with this technique, including implications on the intervertebral disc biology, and will need to be investigated in the future.

## Conclusion

In conclusion, AIS is a complex and relatively common disease without a clear understanding of the etiology behind it. If identified in the earlier stages non-operative treatment with bracing can lead to prevention of curve progression and the need for surgical intervention. Thus, we agree with the position statement of AAOS, SRS, POSNA, and AAP and endorse school screening measures. Once AIS is identified, the radiation burden can be substantial due to repeat x-rays in these adolescents. For this reason, the EOS system has begun to gain popularity. Additionally, this system will allow 3D reconstruction of curves which can assist with pre-operative planning if the curves do progress. Posterior spinal fusion remains a highly successful, safe, and reliable surgical treatment. New Developments in alternative surgical techniques could have a promising future, especially for skeletally immature patients. Further research is needed, however, before widespread adoption of these techniques.

## Data Availability

No datasets were generated or analysed during the current study.
